# Sensory Adaptation to Chemical Cues by Vomeronasal Sensory Neurons

**DOI:** 10.1523/ENEURO.0223-18.2018

**Published:** 2018-08-13

**Authors:** Wen Mai Wong, Maximilian Nagel, Andres Hernandez-Clavijo, Simone Pifferi, Anna Menini, Marc Spehr, Julian P. Meeks

**Affiliations:** 1 Department of Neuroscience, University of Texas Southwestern Medical Center, Dallas, TX 75390; 2 Department of Chemosensation, Institute for Biology II, Rheinisch-Westfälische Technische Hochschule Aachen University, Aachen D-52074, Germany; 3 Neurobiology Group, SISSA, Scuola Internazionale Superiore di Studi Avanzati, Trieste 34136, Italy

**Keywords:** adaptation, calcium imaging, chemical senses, electrophysiology, olfaction, vomeronasal system

## Abstract

Sensory adaptation is a source of experience-dependent feedback that impacts responses to environmental cues. In the mammalian main olfactory system (MOS), adaptation influences sensory coding at its earliest processing stages. Sensory adaptation in the accessory olfactory system (AOS) remains incompletely explored, leaving many aspects of the phenomenon unclear. We investigated sensory adaptation in vomeronasal sensory neurons (VSNs) using a combination of *in situ* Ca^2+^ imaging and electrophysiology. Parallel studies revealed prominent short-term sensory adaptation in VSNs upon repeated stimulation with mouse urine and monomolecular bile acid ligands at interstimulus intervals (ISIs) less than 30 s. In such conditions, Ca^2+^ signals and spike rates were often reduced by more than 50%, leading to dramatically reduced chemosensory sensitivity. Short-term adaptation was reversible over the course of minutes. Population Ca^2+^ imaging experiments revealed the presence of a slower form of VSN adaptation that accumulated over dozens of stimulus presentations delivered over tens of minutes. Most VSNs showed strong adaptation, but in a substantial VSN subpopulation adaptation was diminished or absent. Investigation of same- and opposite-sex urine responses in male and female VSNs revealed that adaptation to same-sex cues occurred at ISIs up to 180 s, conditions that did not induce adaptation to opposite-sex cues. This result suggests that VSN sensory adaptation can be modulated by sensory experience. These studies comprehensively establish the presence of VSN sensory adaptation and provide a foundation for future inquiries into the molecular and cellular mechanisms of this phenomenon and its impact on mammalian behavior.

## Significance Statement

Peripheral sensory adaptation is an initial mechanism for experience-driven feedback in the nervous system. In the mammalian accessory olfactory system (AOS), which senses many social chemosignals, peripheral adaptation remains a subject of some debate. We investigated sensory adaptation to natural cues and monomolecular ligands in acute preparations of the vomeronasal organ, finding evidence for short- and long-term adaptation on repeated stimulation at defined intervals. We further tested whether adaptation differed to same-sex versus opposite-sex cues, finding that sensory adaptation was more prominent for same-sex cues in both males and females. These inquiries into AOS adaptation provide a foundation for future inquiries into this phenomenon.

## Introduction

Terrestrial mammals detect a wide array of chemosensory cues using distinct olfactory subsystems, the most prominent of which are the main olfactory system (MOS) and the accessory olfactory system (AOS). The MOS is specialized for the detection of volatile ligands that are able to activate olfactory sensory neurons in the main olfactory epithelium. In the AOS, social cues, including pheromones, dissolve in nasal mucus and enter the vomeronasal organ (VNO) where they activate vomeronasal sensory neurons (VSNs). These neurons detect social chemosignals via activation of G protein-coupled receptors including members of the V1R, V2R, and formyl peptide receptor families (Dulac and Axel, 1995; Herrada and Dulac, 1997; Matsunami and Buck, 1997; Ryba and Tirindelli, 1997; Liberles et al., 2009; Rivière et al., 2009). Activation of VSNs and downstream brain regions controls the expression of several behaviors and physiologic phenomena, including territorial aggression, pregnancy block, and predator avoidance (Wysocki et al., 1982; Brennan et al., 1990; Stowers et al., 2002; Papes et al., 2010; Kaur et al., 2014). As such, understanding VSN physiology is essential for understanding AOS chemosensation and its impacts on mammalian physiology and behavior.

An important feature of the MOS is its capacity for sensory adaptation, a process which results in reduced sensitivity to odorants in their continued presence (for review, see Menini, 1999; Zufall and Leinders-Zufall, 2000). Though adaptation in olfactory sensory neurons has been well established and characterized (Kurahashi and Menini, 1997; Munger et al., 2001; Song et al., 2008; Stephan et al., 2011), there remains some debate about the phenomenon in VSNs (Holy et al., 2000; Nodari et al., 2008; Spehr et al., 2009; Arnson and Holy, 2011). Studies showing little evidence for adaptation used extracellular single- and multi-unit action potential recordings from VSNs (Holy et al., 2000; Nodari et al., 2008; Arnson and Holy, 2011), while those finding evidence for adaptation used extracellular field potential recordings and patch clamp techniques (Spehr et al., 2009). The differing conclusions may reflect the different methodologies used (e.g., multielectrode array recordings vs local field potentials, etc.), stimulus panels (e.g., dilute urine vs monomolecular steroid ligands) or other unknown variables.

In this study, we present data on VSN sensory adaptation from three individual laboratories using different physiological approaches, stimuli, and adaptation-testing paradigms. Each dataset independently presents evidence that most VSNs undergo pronounced sensory adaptation on repeated exposure to the same stimuli over varying time courses. Short-term adaptation was induced at interstimulus intervals (ISIs) less than ∼30 s in most VSNs. However, a subset of VSNs proved resistant to adaptation even at short ISIs. Hours-long population Ca^2+^ imaging experiments revealed additional slow VSN adaptation that was sensitive to the timing of stimuli but not the total stimulus exposure or imaging time. Finally, VSN recordings from male and female mice showed differential levels of adaptation to same- and opposite-sex urine, suggesting that long-term sensory exposure increases the prevalence of short-term VSN adaptation. Cumulatively, these results confirm that VSN adaptation occurs on both short and long time scales, indicating that the AOS, like the MOS, utilizes sensory adaptation to tune its physiological responsiveness to chemosensory input over time.

## Materials and Methods

### Animals

Animal procedures for confocal Ca^2+^ imaging experiments were approved by local authorities and in compliance with European Union Legislation (Directive 2010/63/EU) and recommendations by the Federation of European Laboratory Animal Science Associations (FELASA). Animal procedures for volumetric Ca^2+^ imaging experiments were approved by the Institutional Animal Care and Use Committee at the University of Texas Southwestern Medical Center. Animal procedures for electrophysiological experiments were handled in accordance with the guidelines of the Italian Animal Welfare Act and European Union guidelines on animal research, under a protocol approved by the ethics committee of Scuola Internazionale Superiore di Studi Avanzati (SISSA). Confocal Ca^2+^ imaging experiments were performed in wild-type C57BL/6 mice. Volumetric Ca^2+^ imaging experiments were performed with *OMP^tm4(cre)Mom^/*J knock-in mice (*OMP-Cre* mice; The Jackson Laboratory stock #006668) mated to *Gt(ROSA)26Sor^tm96(CAG-GCaMP6s)Hze^*/J mice (Ai96 mice; The Jackson Laboratory stock #024106). These mice express the genetically encoded Ca^2+^ indicator GCaMP6s in VSNs (*OMP-Cre*^+/-^, Ai96^+/-^ double transgenic mice), further referred to as OMPxAi96 mice. Electrophysiological experiments were performed in wild-type C57BL/6 mice.

### Chemicals, solutions, and stimuli presentation

For confocal Ca^2+^ imaging, the following solutions were used (300 mOsm, adjusted with glucose; pH 7.3, adjusted with NaOH): HEPES buffered extracellular solution containing 145 mM NaCl, 5 mM KCl, 1 mM CaCl_2_, 1 mM MgCl_2_, and 10 mM HEPES (S_1_); oxygenated (95% O_2_, 5% CO_2_) extracellular solution containing 125 mM NaCl, 25 mM NaHCO_3_, 5 mM KCl, 1 mM CaCl_2_, 1 mM MgSO_4_, and 5 mM N,N-bis(2-hydroxyethyl)-2-aminoethanesulfonic acid (S_2_); and elevated K^+^ solution containing 100 mM NaCl, 50 mM KCl, 1 mM CaCl_2_, 1 mM MgCl_2_, and 10 mM HEPES (S_3_). Free Ca^2+^ and Mg^2+^ concentrations were calculated using WEBMAXC STANDARD (available at http://www.stanford.edu/∼cpatton/webmaxcS.htm). If not stated otherwise, chemicals were purchased from Sigma. For sensory stimulation, urine samples from ≥10 individuals were pooled and immediately frozen (-20°C) as ready-to-use aliquots. Before experiments, aliquots were thawed and diluted 1:100 in S_1_ (Spehr et al., 2009). Stimuli were applied for 10 s from air pressure-driven reservoirs via an 8-in-1 multi-barrel “perfusion pencil” (Science Products). Changes in focal superfusion (Veitinger et al., 2011) were software controlled and synchronized with data acquisition by TTL input to 12V DC solenoid valves using a TIB 14S digital output trigger interface (HEKA Elektronik).

For volumetric Ca^2+^ imaging, cholic acid (CA) and deoxycholic acid (DCA) stocks (20 mM) were prepared in methanol and stored at -20°C. For experiments, bile acid stocks were further diluted to 10 µM in Ringer’s solution (S_4_) containing 115 mM NaCl, 5 mM KCl, 2 mM CaCl_2_, 2 mM MgCl_2_, 25 mM NaHCO_3_, 10 mM HEPES, and 10 mM glucose. The final methanol concentration in both bile acid test stimuli and control Ringer’s saline was 0.5%. Stimuli were applied for 15 s using an air pressure-driven reservoir via a 16-in-1 multi-barrel perfusion pencil (Automate Scientific). Stimulus delivery was synchronized with image acquisition using custom software (Holekamp et al., 2008; Turaga and Holy, 2012).

For electrophysiological recordings, urine from adult (more than two months, both sexes) C57BL/6 mice were filtered separately (0.2-µm filter) and frozen at -80°C. Before use, male and female urines were mixed in a 1:1 ratio and the mixture was diluted 1:50 in extracellular solution (S_5_) containing 120 mM NaCl, 20 mM NaHCO_3_, 3 mM KCl, 2 mM CaCl_2_, 1 mM MgSO_4_, 10 mM HEPES, and 10 mM glucose; pH 7.4. As urine contains urea and potassium ions, which could potentially cause neurons to fire by direct membrane depolarization, artificial urine diluted 1:50 was used as a control. Artificial urine (S_6_) contained 100 mM NaCl, 40 mM KCl, 20 mM NH_4_OH, 4 mM CaCl_2_, 2.5 mM MgCl_2_, 15 mM NaH_2_PO_4_, 20 mM NaHSO_4_, 333 mM urea; pH 7.4 (NaOH; Holy et al., 2000).

### Tissue preparation

For confocal Ca^2+^ imaging, acute coronal VNO slices of C57BL/6 mice (including four female and five male mice; three to nine months), were prepared as previously described (Hagendorf et al., 2009; Cichy et al., 2015). Briefly, mice were sacrificed by brief exposure to CO_2_ followed by cervical dislocation and decapitation. The lower jaw and palate were removed. The VNO was dissected, embedded in 4% low-gelling temperature agarose, placed in ice-cold oxygenated S_2_, and coronal slices (150 μm) were cut on a VT1000S vibrating microtome (Leica Biosystems). Slices were transferred to a submerged, chilled, and oxygenated storage chamber with circulating S_2_ until use.

Volumetric Ca^2+^ imaging experiments were performed in OMPxAi96 mice (including three female and four male mice; 1.5–3 months). Following deep isoflurane anesthesia, VNOs were dissected out and placed into ice-cold oxygenated S_4_. Under a dissection microscope (Leica Microsystems) the vomeronasal pump was surgically removed from the vomeronasal epithelium. The vomeronasal epithelium was then adhered to a small piece of nitrocellulose paper and secured in a recording chamber using a custom clip.

For electrophysiological recordings, acute coronal slices of mouse VNO were prepared as previously described (Shimazaki et al., 2006; Dibattista et al., 2008). In brief, the VNO was removed from C57BL/6 mice (including three female and three male mice; two to three months) and transferred to ice-cold S_5_. After the capsule and all cartilaginous tissues were carefully removed, the two halves of the VNO were isolated from the vomer bone and embedded in 3% low-melting temperature agarose once it had cooled to 38°C. On solidification, a vibrating microtome (Vibratome 1000 Plus Sectioning System, Warner Instruments) was used to cut coronal slices of 200- to 250-µm thickness in ice-cold oxygenated S_5_. Slices were then left to recover for at least 30 min in ice-cold oxygenated S_5_ before electrophysiological experiments were initiated.

### Confocal Ca^2+^ imaging

For Ca^2+^-sensitive dye loading, acute coronal VNO slices were incubated (60 min; 5°C) in circulating S_2_ (storage chamber) containing Cal-520/AM (4.5 µM; Biomol) and 0.0005% pluronic F-127 (20% solution in DMSO; Thermo Fisher Scientific). After washing five times (S_2_), VNO slices were transferred to a recording chamber (Luigs & Neumann) on an upright fixed-stage scanning confocal microscope (TCS SP5 DM6000CFS, Leica Microsystems) equipped with a 20×/1.0 NA water immersion objective (HCX APO L, Leica Microsystems) and infrared-optimized differential interference contrast (IR-DIC) optics. Bath solution (S_2_) was continuously exchanged (∼5 ml/min; gravity flow). Cal-520 was excited using the 488-nm line of an argon laser. Changes in cytosolic Ca^2+^ were monitored over time at 1.0-Hz frame rates. Neurons were stimulated at decreasing ISIs of 180, 60, and 30 s.

### Volumetric Ca^2+^ imaging

The vomeronasal epithelium was imaged using a custom objective-coupled planar illumination (OCPI) microscope (Holekamp et al., 2008). In brief, a light sheet was formed using a lens relay (Tower Optical; Edmund Optics), within a miniaturized light sheet-forming tube. A custom fiber optic (OZ Optics) relayed 491-nm laser light (Coherent) and collimated the beam before the miniaturized lens relay. A custom, tunable coupler was used to orient the light sheet at 90° relative to a 20×, 0.5 NA objective lens (Olympus). The adjustable coupler was used to align the light sheet with the focal plane of the objective. The objective lens was connected to a piezoelectric positioner (Physik Instrumente, L.P.) to synchronously adjust the light sheet and focal plane of the objective, resulting in optical sectioning.

Each image stack consisted of 51 frames, spanning ∼700 µm in the lateral direction, 250–400 µm in the axial direction, and ∼150 µm in depth. Custom software synchronized imaging with a computer-controlled stimulus delivery device (Automate Scientific). Ringer’s saline (S_4_) was continuously applied to the epithelium at a rate of ∼2 ml/min. All stimuli were diluted in S_4_ and applied at the same flow rate through the same port. Three-dimensional image stacks were acquired once every 3 s, and stimuli were applied for five consecutive stacks (∼15 s) with varying ISIs. Stimulation patterns are described in Results.

### Loose-patch extracellular recordings

Acute VNO slices were placed in a recording chamber, continuously perfused with oxygenated S_5_ and viewed with an upright microscope (Olympus BX51WI) by IR-DIC optics with water immersion 20× or 60× objectives. Stimuli were delivered through an 8-into-1 multi-barrel perfusion pencil connected to a ValveLink8.2 pinch valve perfusion system (Automate Scientific). A 5-s urine pulse was delivered, followed by a second identical pulse at interpulse intervals ranging from 5 to 60 s. The time between each double-pulse experiment was ≥2 min. To measure firing activity in intact VSNs, extracellular action potential-driven capacitive currents were measured in the on-cell loose-patch configuration (Ferrero et al., 2013; Lorenzon et al., 2015; Ackels et al., 2016; Pietra et al., 2016). Patch pipettes (3- to 6-MΩ resistance), pulled from borosilicate capillaries (WPI) with a PC-10 puller (Narishige), were filled with extracellular solution. Seal resistances of 40–100 MΩ were obtained on the soma of VSNs. Recordings were made in voltage-clamp mode with a holding potential of 0 mV using a MultiClamp 700B amplifier controlled by Clampex 10 via a Digidata 1440 (Molecular Devices). Data were low-pass filtered at 2 kHz and sampled at 5 kHz. Experiments were performed at room temperature.

### Analysis of confocal imaging data

All data were obtained from independent experiments performed on at least three days using at least three different animals. Individual numbers of cells/experiments are denoted in the figure legends. Unless stated otherwise, results are presented as means ± SEM. Data were analyzed offline using Leica LAS AF 2.4 (Leica Microsystems), IGOR Pro 7.0 (WaveMetrics), ImageJ 1.51n (Wayne Rasband, National Institutes of Health) and Excel (Microsoft) software. Statistical analyses were performed using paired *t* tests, Dunnett test, or one-way ANOVA with Tukey’s HSD *post hoc* test. Tests and corresponding *p* values that report statistical significance (≤0.05) are individually specified in figure legends. In fluorescence imaging experiments, regions of interest (ROIs) were defined to encompass all depolarization-sensitive (S_3_) somata per field of view based on DIC imaging of cell morphology at rest. Time-lapse fluorescence images were movement corrected with the StackReg/Rigid Body transformation plugin (Thevenaz et al., 1998) in ImageJ. Changes in relative fluorescence intensity were calculated as ΔF/F and measured in arbitrary units. Neurons were classified as “responsive” when showing stimulus-dependent Ca^2+^ elevations in somata according to the following three criteria (Fluegge et al., 2012): (1) exposure to high extracellular K^+^ concentrations (50 mM; S_3_) induced a robust Ca^2+^ transient; (2) the peak intensity value of at least one urine presentation exceeded the average prestimulation baseline intensity plus three standard deviations for at least 3 s [I_resp_ > I_baseline_ + 3 × SD(I_baseline_)]; and (3) the increase in fluorescence intensity was observed within the time window of stimulus application. Every responsive cell was visually inspected and neurons showing potentially unspecific responses (e.g., because of high spontaneous activity) were excluded.

### Analysis of volumetric Ca^2+^ imaging data

All data analysis was performed in MATLAB using custom software similar to (Turaga and Holy, 2012; Hammen et al., 2014). In brief, image stacks were registered using a two-stage algorithm, essentially a rigid translation followed by a nonrigid warping. Custom MATLAB software calculated the relative change in GCamP6s intensity (ΔF/F) for every image voxel by subtracting the mean voxel intensity in three consecutive prestimulus stacks from the mean voxel intensity in three stacks during the stimulus delivery, then dividing the resulting value by the prestimulus intensity. ΔF/F “maps” for each stimulus were used to identify well-registered VSNs that reliably responded to stimulation. In some cases in which residual “drift” was apparent post-registration, ROIs were realigned with the somata at regular time intervals to avoid false negative responses. Following volumetric ROI selection, the mean voxel intensity for each ROI was calculated for every image stack in the experiment (∼1500 stacks), generating an nROIs × nStacks matrix of fluorescence intensity. This intensity matrix was used to calculate ΔF/F for every ROI and every stimulus application.

Slow, interblock VSN adaptation was calculated by normalizing ΔF/F on the first trials of each stimulus in each block to the maximal ΔF/F response across all trials. Fast, intrablock VSN adaptation was calculated using the following formula:

$$I_{intra(b)}=\frac{{\left(\Delta F/F\right)}_{\left(b,t3\right)}-{\left(\Delta F/F\right)}_{\left(b,t1\right)}}{{\left(\Delta F/F\right)}_{\left(b,t1\right)}+0.1}$$

Where ${\left(\Delta F/F\right)}_{\left(b,t3\right)}$ is the ΔF/F response of the third trial (t3) of blockb and ${\left(\Delta F/F\right)}_{\left(b,t1\right)}$ is the ΔF/F response of the first trial (t1) of blockb. The 0.1 offset to the denominator is added to avoid divide by zero errors. All data points with error bars reflect mean ± SEM.

Discriminability (d’) of “CA-only” and DCA-only VSN populations across blocks/trials was calculated similarly to (Davison and Katz, 2007):

$${d^{'}}_{\left(b,t\right)}=\frac{\left|{mean\left(\Delta F/F\right)}_{(DCA-only)\left(b,t\right)}-mean{\left(\Delta F/F\right)}_{(CA-only)\left(b,t\right)}\right|}{\sqrt{\frac{{std{\left(\Delta F/F\right)}_{(DCA-only)\left(b,t\right)}}^{2}+{std{\left(\Delta F/F\right)}_{(CA-only)\left(b,t\right)}}^{2}}{2}}}$$

where $mean{\left(\Delta F/F\right)}_{(DCA-only)\left(b,t\right)}$ represents the mean within-cell normalized ΔF/F responses to 10 µM DCA on block b and trial t. The term $std{\left(\Delta F/F\right)}_{(DCA-only)\left(b,t\right)}$ is the standard deviation of within-cell normalized in normalized ΔF/F responses to 10 µM DCA on block b and trial t.

### Analysis of electrophysiological data

IgorPro 6.7 software (WaveMetrics) and Clampfit (Molecular Devices) were used for data analysis and figure preparation. Data are presented as mean ± SEM. Because most of the data were not normally distributed (Shapiro–Wilk test), statistical significance was determined using Friedman’s test followed by Tukey–Nemenyi test; *p* < 0.05 was considered statistically significant. For spike detection, recordings were filtered offline with a high-pass filter at 2 Hz to eliminate slow changes in the baseline. Individual spikes were identified by a self-written threshold detection procedure and confirmed by shape inspection. The spontaneous firing activity of each VSN was evaluated before urine application. A VSN was considered responsive to urine if (1) the average spike frequency during the urine pulse was higher than the average spontaneous spike frequency plus two standard deviations, and (2) it was not responsive to artificial urine.

## Results

### VSNs demonstrate short-term adaptation to repeated presentations of mouse urine

**Figure 1.**
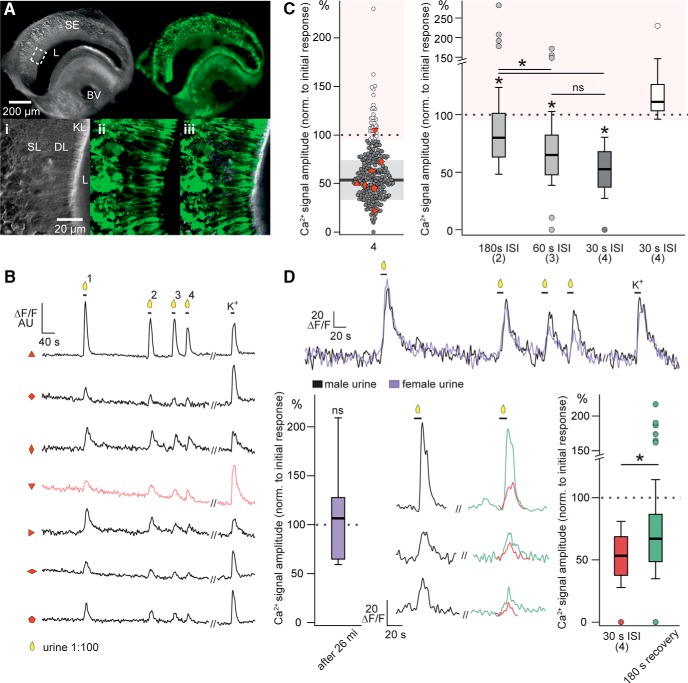
The majority of mouse VSNs display sensory adaptation on repeated stimulation with diluted urine. ***A***, Loading of sensory neurons in acute VNO slices with a synthetic Ca^2+^ indicator. Top, Low-magnification DIC (left) and epi-fluorescence image (right) of a VNO section bulk-loaded with Cal-520/AM. Bottom, High-magnification images of a region of the sensory epithelium (white box in top image). Confocal DIC (left) and fluorescence (middle) images are merged (right) to show dye loading of sensory neurons. ***B***, Representative original recordings of cytosolic Ca^2+^ signals in different VSN somata in response to diluted urine (1:100; 10 s) and elevated extracellular potassium (K^+^; 50 mM; 10 s). The integrated relative fluorescence intensities (ΔF/F) in user-defined ROIs are displayed in arbitrary units and viewed as a function of time. Neurons are stimulated at decreasing ISI of 180 s (1 → 2), 60 s (2 → 3), and 30 s (3 → 4). Black traces represent VSNs that undergo sensory adaptation of variable degree. Red trace shows a neuron that displays no adaptation. ***C***, left, Scatter dot plot depicting relative Ca^2+^ signal amplitudes recorded from a total of 259 VSNs in response to stimulation 4 [data points show signal strength as percentage of the initial response amplitude (1)]. Data are categorized as indicative of adaptation (<95%; gray; *n* = 227) or the lack thereof (≥95%; white; *n* = 32). Data points marked by different red symbols correspond to original recordings shown in ***B***. Solid horizontal line and gray shadow indicate mean ± SD of adapting VSNs. Right, Box plots illustrating the Ca^2+^ signal amplitudes evoked by stimulations 2 (180 s ISI), 3 (60 s ISI), and 4 (30 s ISI). Data are shown as percentage of the response to initial stimulation (1). Median values (horizontal lines), the interquartile ranges (height of the box), and the minimum and maximum values (whiskers) are plotted. Circles depict values that were >1.5 times the interquartile range from the lower or upper quartile. White box (right) corresponds to the VSNs categorized as not adapting. ***D***, top, Original recording from a representative VSN consecutively challenged with male (black trace) and female (purple trace) urine. Interval between recordings 26 min. Fluorescence intensity (ΔF/F) is plotted as a function of time. Stimulation paradigm as in ***B***. Bottom, Box plot (left; *n* = 27) quantifying and comparing the Ca^2+^ signal amplitudes evoked by the initial stimulations with diluted male and female urine, respectively. Note that sensitivity is fully restored after 26 min. Middle/right, Original traces and box plot quantification illustrating initial responses (black traces), responses to stimulation 4 (30 s ISI; red traces and box), and responses to a fifth stimulation after an additional 180 s recovery period (green traces and box). Asterisk (*) indicates statistical significance, *p* < 0.01 (one-way ANOVA in ***C***; paired *t*-test in ***D***). BV, blood vessel; DL, dendritic layer; KL, knob layer; L, lumen; SE, sensory epithelium; SL, soma layer; VNO, vomeronasal organ.

As sensory adaptation in the VNO is still subject to debate, we first asked whether repeated sensory stimulation of VSNs resulted in robust repetitive responses or, by contrast, if VSNs revealed signs of sensory adaptation on repeated stimulus exposure. With the goal of resolving previous discrepancies, we designed experiments that allowed us to investigate VSN adaptation at the individual cell and population levels. We loaded VSNs in acute coronal VNO slices from wild-type C57BL/6J mice with the Ca^2+^-sensitive dye Cal-520/AM. We then performed time-lapse confocal Ca^2+^ imaging while stimulating repetitively with diluted urine samples pooled from either male or female conspecifics of the same C57BL/6J strain (*n* = 6; Fig. 1*A*). We monitored Ca^2+^ concentrations in the somata of all neurons that showed robust transient elevations in cytosolic Ca^2+^ on depolarization (exposure to increased extracellular K^+^). In each experiment, focal stimulus application was repeated four times at decreasing ISIs of 180, 60, and 30 s.

From a total of 259 urine-sensitive VSNs from both male and female mice, we observed a substantially reduced Ca^2+^ response in 227 neurons (87.6%) when signal amplitudes evoked by the fourth stimulation were compared to the initial urine exposure (Fig. 1*C*). Notably, the remaining 32 VSNs (12.4%) did not reveal any reduction in sensitivity. On average, small but significant amplitude reductions were observed at 180-s ISI (85.7 ± 2.2%; one-way ANOVA, *F* = 31.99, *F*c = 2.61, *p* = 0, Tukey’s HSD *p* = 0.0055). On shorter ISIs of 60 and 30 s, we observed more pronounced mean amplitude reductions to 67.5 ± 1.9% (Tukey’s HSD, *p* = 6.18 × 10^−10^) and 53.8 ± 1.4% (Tukey’s HSD, *p* = 2.46 × 10^−12^), respectively. The 32 VSNs that did not show signs of adaptation in this stimulation paradigm showed average Ca^2+^ signal amplitudes in response to the last stimulation that were statistically indifferent as compared to the initial signal (121.0 ± 4.6%; one-way ANOVA, *F* = 0.97, *F*c = 2.67, *p* = 0.407). Notably, a fifth stimulation after an additional 180-s ISI induced a significantly higher mean response amplitude (71.8 ± 2.3%; paired *t* test, *p* = 9.15 × 10^−13^) as compared to the fourth stimulation (Fig. 1*D*), indicating that short-term VSN adaptation is at least partially reversible over the time course of minutes.

In a smaller set of VSNs, we compared Ca^2+^ signal amplitudes evoked by diluted male and female urine. In these experiments, a stimulation-free period of 21.5 min was between the last male stimulus application and the next exposure to female urine. In those 27 VSNs that responded to both male and female stimuli, initial Ca^2+^ signal amplitudes in response to female urine were statistically indistinguishable (110.7 ± 10.4%; paired *t* test, *p* = 0.78) from Ca^2+^ transients evoked the initial exposure to male stimuli (Fig. 1*D*). Combined, these data reveal the presence of pronounced short-term adaptation at the population and individual cell levels, especially for short ISIs, but also indicate that VSNs can retain stimulus sensitivity over long experimental time courses. This latter finding is consistent with previous studies that found no appreciable adaptation during long experiments involving sparse stimulation with monomolecular sulfated steroid ligands (Nodari et al., 2008; Arnson and Holy, 2011).

### VSNs demonstrate long-term and short-term adaptation to monomolecular ligands

**Figure 2.**
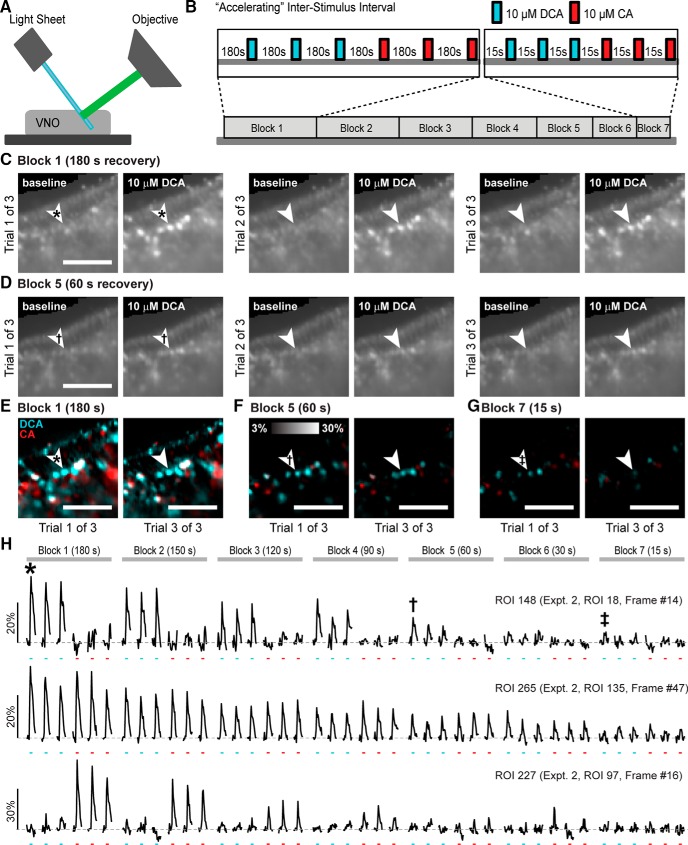
Evaluating sensory adaptation to monomolecular AOS ligands with population VSN Ca^2+^ imaging. ***A***, VNO imaging setup using OCPI (light sheet) microscopy, which enables volumetric imaging of thousands of VSNs in the intact vomeronasal epithelium. ***B***, Experimental design for accelerating protocol of stimulus acquisition. Two monomolecular ligands, DCA and CA, were applied in blocks of six trials (three each for DCA and CA) with the noted recovery periods within each block. ***C***, ***D***, Example images taken from a single frame of a 51-frame z-stack showing the responses of VSNs to repeated stimulation with 10 µM DCA in “Block 1” (180 s recovery, **C**) and “Block 5” (60 s recovery, ***D***). Arrowheads mark the position of a DCA-responsive cell. Scale bar: 100 µm. Symbols refer to derivative images in ***E–G*** and traces shown in ***H***. ***E–G***, Normalized change in fluorescence (ΔF/F) of VSNs on trial 1 of 3 (left) and trial 3 of 3 (right) in Block 1 (180 s recovery, ***E***), Block 5 (60 s recovery, ***F***), and Block 7 (15 s recovery, ***G***). Arrowheads mark the same cell as in ***C***, ***D***. ***H***, Across-trial responses of a DCA-selective cell (top, same as the cell shown in ***C****–****G***), a DCA- and CA-responsive cell (middle), and a CA-selective cell (bottom). Symbols refer to the responses noted in ***C****–****G***.

Urine contains many AOS ligands, raising the possibility that the variability of VSN adaptation may, at least in part, reflect the activation of VSNs expressing many receptors (i.e., different VSN “types”). Therefore, we next examined VSN adaptation to monomolecular ligands. Specifically, we investigated VSN adaptation to the bile acids CA and DCA, two natural AOS ligands found in feces (Doyle et al., 2016). To test VSN adaptation to these monomolecular bile acids, we used volumetric VSN Ca^2+^ imaging via OCPI microscopy (Holekamp et al., 2008). We imaged the intact VNO epithelium in OMPxAi96 mice, which express the genetically encoded calcium indicator GCaMP6s in VSNs (Li et al., 2004; Madisen et al., 2015; Fig. 2*A*). Using a computer-controlled fast stimulation device we stimulated the VNO epithelium with DCA and CA, one at a time, with an “accelerating” protocol in which ISIs were systematically decreased from 180 to 15 s in blocks of three trials per ligand (Fig. 2*B*). At the transition between blocks, the ISI for the upcoming block was used (e.g., between the 180- and 150-s block in the accelerating protocol the ISI was 150 s). Using this approach, we were able to track individual VSNs within volumetric ROIs for 1500 or more image stacks (>4500 s; Fig. 2*C–H*). Consistent with previous studies in the accessory olfactory bulb (Doyle et al., 2016), we observed differential tuning to CA and DCA at 10 µM (Fig. 2*E–H*). In the accelerating protocol, we observed a steady decline in VSN response intensity (ΔF/F) to each monomolecular ligand across blocks.

**Figure 3.**
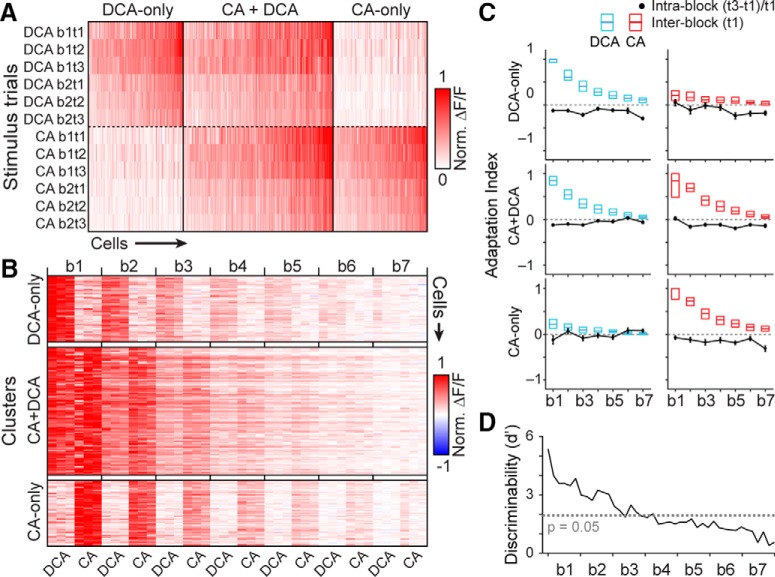
VSN responses to monomolecular bile acid ligands in the accelerating intertrial interval paradigm. ***A***, Clustering of VSN responses to 10 µM bile acids reveals three populations: those that respond to DCA-only, CA and DCA (CA + DCA), and CA-only. Each column represents ΔF/F for a single VSN across six trials of each stimulus (all trials from blocks 1 and 2). Includes responses from 486 VSNs across four tissues (four animals). Two experiments presented DCA first and two presented CA first. ***B***, Normalized response patterns of clustered VSNs across all blocks/trials. Shown are VSNs from two experiments in which DCA was presented first. Each row is a VSN and each column a single trial. Blocks are noted by vertical ticks. ***C***, Adaptation across and within each stimulus block, arranged by cluster. Box plots indicate the median (dark line) and 25–75% interquartile range of the per-VSN normalized response magnitude of the first trial (t1) of each block. Solid black lines/symbols indicate the degree of intrablock adaptation ([t3-t1]/[t1+offset]). ***D***, Reduction in cluster discriminability (d’) resulting from inter- and intratrial adaptation.

We next investigated whether the apparent accumulation of sensory adaptation was modulated by sensory responsiveness to CA and DCA at the 10 µM concentration. We took advantage of the observation that subsets of VSNs are selective for 10 µM DCA (DCA-only), selective for 10 µM CA (CA-only), or nonselectively activated by both stimuli (“CA+DCA”; Figs. 2*E–H*, 3*A* ) to independently evaluate adaptation in each functionally-defined subset. We assigned each VSN to one of three clusters based on their pattern of responsiveness across the initial two blocks of stimulation (including six trials of each monomolecular bile acid at 10 µM; Fig. 3*A*). Pooling across accelerating experiments, we identified 486 ROIs that reliably responded to stimulation across the initial two blocks. Of these ROIs, 135 (27.8%) were selectively activated by 10 µM DCA, 136 (28.0%) were selectively activated by 10 µM CA, and 215 (44.2%) were activated by both 10 µM DCA and 10 µM CA (Fig. 3*A*). We observed no difference in the rate of slow, cumulative sensory adaptation across these clusters (Fig. 3*B*,*C*), despite the fact that VSNs in the CA+DCA cluster were active on every trial whereas each of the selective clusters were only activated in half of the trials in each block. In the initial blocks, the discriminability index, which quantifies the separation in a population of responses (Davison and Katz, 2007), showed a high degree of discriminability between VSNs in the DCA-only and CA-only clusters (Fig. 3*D*). The net effect of the slow, cumulative sensory adaptation in the accelerating ISI protocol was a steady decrease in the discriminability index between these initially well-separated clusters. By the time the shortest intertrial intervals were tested (block 7), the discriminability between these clusters was effectively abolished (Fig. 3*D*). Importantly, our ability to evaluate faster adaptation (e.g., that seen at the shortest ISIs in Fig. 1) with this paradigm was limited by the apparent slow adaptation.

**Figure 4.**
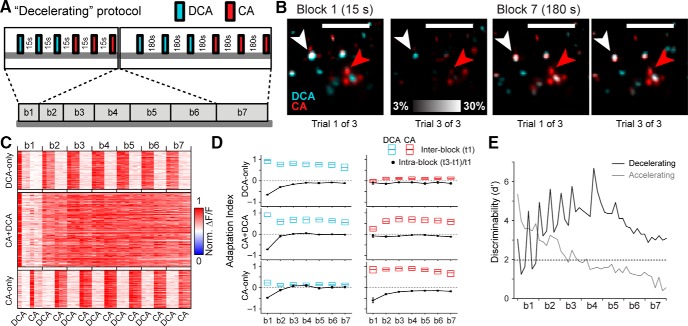
VSN responses to monomolecular bile acid ligands in the decelerating intertrial interval paradigm. ***A***, Stimulus delivery design. ***B***, Example ΔF/F images showing the responses of VSNs to repeated stimulation with 10 µM DCA and 10 µM CA in “Block 1” (15 s recovery, left) and “Block 7” (180 s recovery, right). White and red arrowheads mark VSNs in the CA+DCA and CA-only clusters, respectively. ***C***, Normalized ΔF/F responses of clustered VSNs across blocks/trials. Shown are VSNs from three experiments in which DCA was presented first. Note that cross-adaptation is largely restricted to the CA+DCA cluster. ***D***, Interblock and intrablock adaptation, arranged by cluster. ***E***, In stark contrast to the accelerating protocol (gray line), discriminability between the DCA-only and CA-only clusters in the decelerating protocol (black line) remained high except in trials separated by 15 s.

The observed pattern of slow, cumulative adaptation suggested that VSNs may experience sensory adaptation on a time course much longer than investigated in previous studies. However, this slow adaptation may have instead arisen, in part or in whole, because of technical confounds, for example GCaMP6s photobleaching, VSN phototoxicity, or slow tissue deterioration in the tissue imaging chamber. To determine if these latter confounds contributed to (or caused) slow adaptation, we designed a “decelerating” ISI protocol that matched the total time of ligand exposure, stimulation, the specific ISIs used, and the total exposure to light sheet illumination. We accomplished this by simply flipping the order of ISI blocks, starting with the 15-s ISI block and increasing the ISIs until the 180-s block (Fig. 4*A*). Here, in the absence of slow adaptation, we readily observed strong adaptation of ΔF/F responses at the initial 15-s ISI block (Fig. 4*B*). This fast adaptation was strong enough to degrade cluster assignments including this block, so we clustered VSN responses into DCA-only, CA-only, and CA+DCA categories based on the response patterns during blocks 3 and 4 of the decelerating stimulus paradigm (DCA-only: 103/336, 30.6%; CA-only: 116/336, 34.6%; CA+DCA: 117/336, 34.8%). Evaluating the sensory adaptation patterns in the decelerating profile revealed the presence of strong sensory adaptation at the fastest ISIs (Fig. 4*C*,*D*), consistent with the adaptation seen at fast time scales in response to mouse urine (Fig. 1). Importantly, VSNs in the decelerating ISI protocol showed no evidence of the slow, cumulative adaptation seen in the accelerating paradigm. Discriminability between the DCA-only and CA-only populations in the decelerating protocol showed degradation only in the blocks showing fast adaptation (15-s ISI block) and remained high as ISIs extended toward the 180-s block (Fig. 4*E*). These results indicate that the slow, cumulative adaptation observed in the accelerating ISI protocol does not arise due to photobleaching, phototoxicity, or tissue degradation, but is instead a result of the repeated stimulus exposure and steadily decreasing ISIs.

### VSNs demonstrate short-term spike frequency adaptation to mouse urine

**Figure 5.**
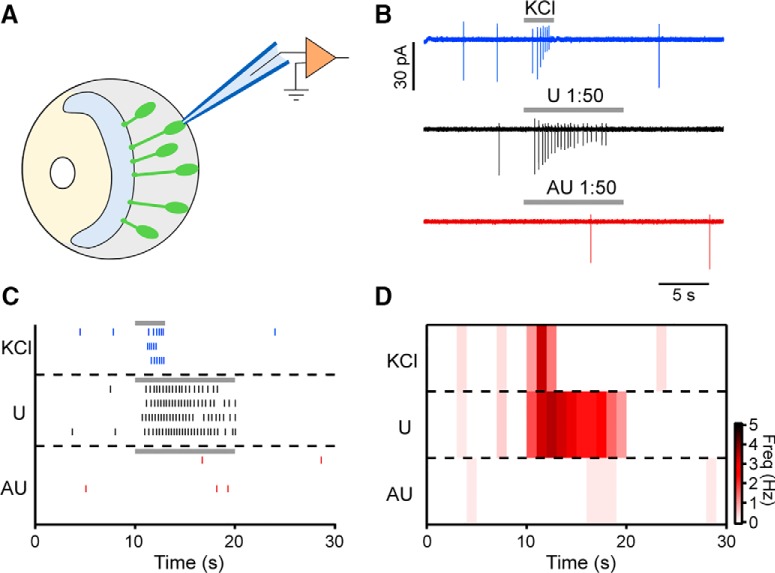
Electrophysiological recordings of evoked firing activity from individual VSNs. ***A***, Schematic representation of a VNO coronal slice and a patch pipette in preparation for electrophysiological recordings from a VSN. ***B***, Representative recordings in the on-cell loose-patch configuration from a single VSN showing the firing activity in response to high K^+^ solution (top), diluted urine (1:50; middle), or diluted artificial urine (1:50; bottom). Time of stimulus presentation is indicated by the top bars. ***C***, Raster plots of firing activity from the same VSN in ***B***. The recovery time between individual stimuli was at least 2 min. ***D***, Heat map showing the average spike frequency of the traces in ***C*** in bins of 1 s. The firing frequency increased in response to urine, or to high K^+^ solution as a positive control, but not in response to artificial urine.

Adaptation in VSN intracellular Ca^2+^ signals may or may not directly relate to spiking activity. To determine if adaptation was also observable in VSN spike frequency (i.e., neuronal output), we performed electrophysiological recordings in the on-cell loose-patch configuration from individual VSNs in acute VNO slices (Fig. 5*A*). Figure 5*B* shows representative responses to the sequence of stimuli used to identify urine-responsive VSNs. A short pulse of high KCl (25 mM) was initially delivered to determine VSN viability. If the neuron showed spike activity in response to high KCl, we tested the response to diluted urine (1:50) and then to artificial urine (1:50). As VSNs often show spontaneous bursting activity (Holy et al., 2000; Arnson and Holy, 2011), each stimulus was repeated at least three times (Fig. 5*C*,*D*) to avoid false positive responses. The representative neuron shown in Figure 5 clearly responded to high KCl and urine with an increase in firing frequency, but it did not show any change in spike activity during artificial urine presentation, indicating that the firing increase to urine presentation was not caused by changes in ion concentration. From a total of 37 VSNs, 18 (48.6%) clearly responded to urine and not to artificial urine.

**Figure 6.**
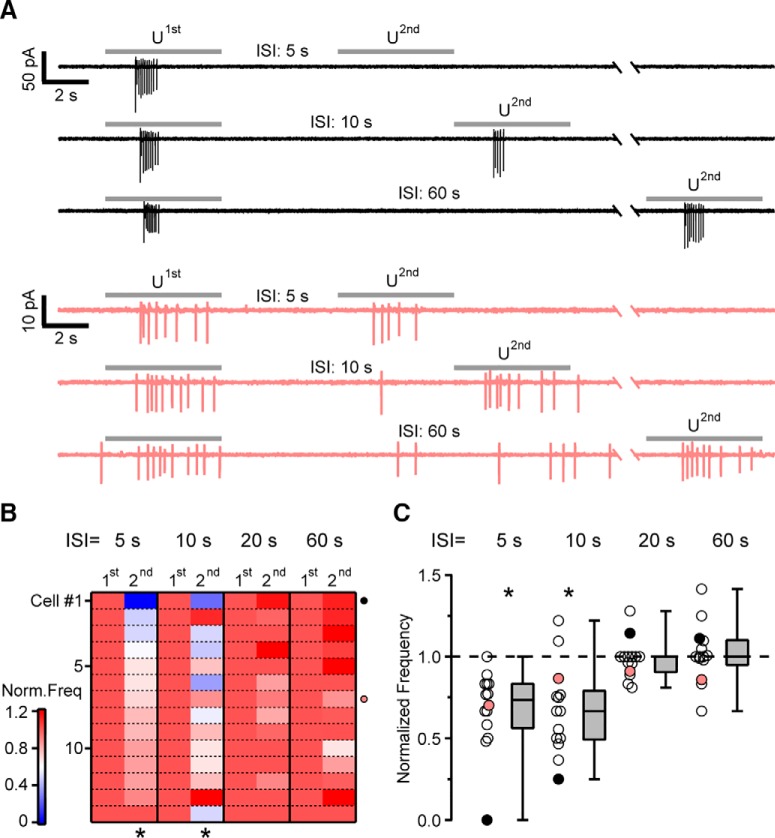
Spike frequency adaptation to repeated mouse urine stimulation of VSNs. ***A***, Representative loose-patch recordings from two individual VSNs in response to double-pulse stimulations with diluted urine (1:50). A 5 s urine pulse was delivered, followed by a second identical pulse with ISIs ranging from 5 to 60 s. The recovery time between each double-pulse experiment was at least 2 min. In black, a urine-responsive VSN did not fire in response to a second pulse of urine after 5 s, showed a reduced firing frequency to the second urine pulse at 10 s ISI, and a recovery at 60 s ISI. In pink, another VSN showing a smaller reduction in firing frequency at 5- and 10-s ISIs compared to the previous VSN. ***B***, Heat map of normalized mean firing frequency from double-pulse urine stimulations at different ISIs for 14 VSNs. The mean frequency during the second urine pulse was normalized to the mean frequency evoked by the first pulse. VSNs shown in ***A*** are indicated by black and green circles. ***C***, Box and scatter plots of the normalized frequency at different ISIs. Each dot represents an individual VSN at a given ISI. In the box plot horizontal lines represent the median, lower and upper box boundaries represent the first and third quartile, respectively, and upper and lower whiskers represent the 5th and 95th percentile. Statistics: Tukey–Nemenyi test after Friedman test: *p < 0.01.

Once we established that a given VSN responded to urine, we proceeded to evaluate the properties of adaptation in response to two repetitive urine pulses separated by ISIs ranging from 5 to 60 s. The recovery time between each repetitive stimulus was at least 2 min. Figure 6*A* shows recordings from a neuron (black traces) displaying a strong decrease in spike frequency to the second urine stimulus applied after 5 or 10 s compared to the spike frequency in response to the first urine stimulus. Spike frequency adaptation steadily diminished as ISIs increased to 20 and 60 s. Some neurons, represented by the neurons highlighted in pink in Figure 6*A*, showed a moderate reduction in spike activity only at the 5-s ISI. To compare the adaptation profile from different neurons, we normalized the average spike frequency in response to the second urine pulse to the value in response to the first urine pulse (Fig. 6*B*,*C*). From a total of 18 urine-responsive VSNs, we could complete the adaptation protocol in 14 VSNs. A quantitative characterization of the urine responses in these neurons shows that spike frequency was significantly different when the second pulse was applied after 5 or 10 s, whereas frequency was not significantly different after 20 or 60 s (Tukey–Nemenyi test after Friedman test *F* = 34.8, p = 1.3 × 10^−7^). Thus, short-term spike frequency adaptation for most VSNs mirrors the time course seen by Ca^2+^ imaging experiments.

### Short-term VSN adaptation to mouse urine is host and stimulus specific

**Figure 7.**
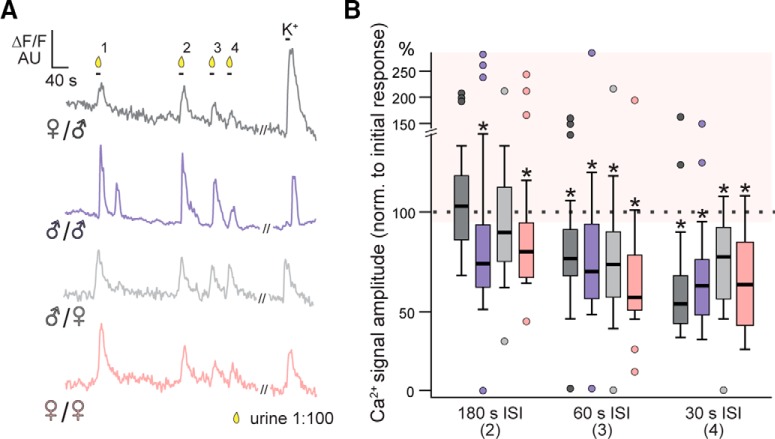
Susceptibility to sensory adaptation is a sex-specific function of the stimulus-recipient combination. ***A***, Representative original recordings of cytosolic Ca^2+^ signals from individual VSN somata in Cal-520/AM-loaded acute VNO slices. The integrated relative fluorescence intensities (ΔF/F) in user-defined ROIs are displayed in arbitrary units and viewed as a function of time. Ca^2+^ transients are evoked by diluted urine (1:100; 10 s) and elevated extracellular potassium (K^+^; 50 mM; 10 s). Temporal stimulation paradigm as in Figure 1. Trace colors indicate specific stimulus-recipient combinations: male VNO/female urine (dark gray), male VNO/male urine (purple), female VNO/male urine (light gray), female VNO/female urine (light red). ***B***, Box plots of Ca^2+^ signal amplitudes (normalized to the initial response) evoked by the 2nd, 3rd, and 4th exposure to urine. Colors denote stimulus-recipient combination as in ***A***: male VNO/female urine (dark gray; *n* = 60), male VNO/male urine (purple; *n* = 46), female VNO/male urine (light gray, *n* = 42), female VNO/female urine (light red, *n* = 79). Note that more pronounced sensory adaptation is apparently evoked by same sex (male-male; female-female) stimulation. Asterisks (*) indicate statistical significance, *p* < 0.05 (Dunnett test).

Ca^2+^ imaging and action potential measurements both revealed the presence of a subset of non-adapting VSNs that are sensitive to components of mouse urine. We hypothesized that VSN adaptation may be (1) dependent on the sex of the animal, (2) the stimulus used, or (3) both. To investigate these questions, we used repeated sensory VSN stimulation in acute Cal-520/AM-loaded VNO slices (Fig. 1*A*) from wild-type C57BL/6J mice. We analyzed Ca^2+^ signal amplitudes evoked by either male or female urine in neurons from male and female mice. These analyses produced several new findings. First, independent of the stimulus–recipient combination, we observed significantly reduced response amplitudes on the third (60-s ISI) and fourth stimulus (30-s ISI) exposure (Fig. 7). Average signal amplitudes were 54.3 ± 2.6% and 50.5 ± 3.4% in female VSNs stimulated with female urine, 69.2 ± 4.8% and 63.7 ± 3.3% in female VSNs stimulated with male urine, 71.2 ± 3.2% and 48.9 ± 2.2% in male VSNs stimulated with female urine, and 70.6 ± 3.4% and 53.6 ± 2.5% in male VSNs stimulated with male urine, respectively. Second, adaptation occurs at longer ISIs when VSNs are challenged with same-sex stimuli. At an ISI of 180 s, stimuli that originate from individuals of the same sex (i.e., male VSNs-male urine and female VSNs-female urine) trigger significant adaptation (female-female: 77.2 ± 3.5%; male-male: 77.2 ± 4.4%), whereas average responses to stimuli that originate from the opposite sex (i.e., male VSNs-female urine and female VSNs-male urine) are essentially unchanged (male-female: 102.2 ± 4.0%; female-male: 86.6 ± 3.9%). Third, at an ISI of 60 s, we observed stronger sensory adaptation when female VSNs were exposed to female urine (54.3 ± 26%), compared to the other three stimulus-recipient combinations. Finally, relief from adaptation differed according to the stimulus-recipient combination. Notably, response recovery is fast when female VSNs are challenged with male urine. A fifth stimulation after an additional ISI of 180 s triggered an average response of 94.74 ± 6.0%. The same period of time did not allow full recovery of sensitivity in the other three stimulus-recipient combinations (female-female: 67.3 ± 4.3%; male-male: 69.9 ± 3.9%; male-female: 59.8 ± 3.2%). Cumulatively, these results suggest that the heterogeneity in VSN adaptation to urinary cues. Moreover, these data suggest that fast VSN adaptation depends on both the sex of the animal and the cues being sensed.

## Discussion

### Short-term VSN adaptation has a time course of seconds and is reversible

Sensory adaptation is a cross-modal phenomenon with important consequences for perception and behavior (for review, see Torre et al., 1995). In the MOS, adaptation begins in the sensory periphery at the level of individual olfactory sensory neurons (for review, see Pifferi et al., 2006). In the AOS, studies of sensory adaptation in VSNs have come to opposing conclusions. Differences in physiologic methods, experimental preparations, and stimulus timing could account for these differences. For example, extracellular single unit recordings from intact VNO epithelial preparations showed only mild within-stimulus spike rate adaptation during constant stimulation for 10 s (Holy et al., 2000; Nodari et al., 2008; Arnson and Holy, 2011). Randomized, interleaved stimulation in such experiments reduced the chances of repeated stimulation with the same odorant in rapid succession. Such protocols have proven beneficial to inquiries into VSN tuning but are not deliberately designed to investigate the presence or absence of short-term VSN adaptation across stimulus presentations.

The data presented here, collected across three independent approaches using different but comparable experimental procedures and stimulus timing protocols, support the conclusion that short-term VSN adaptation occurs over time scales of tens of seconds. This short-term adaptation was observed in response to both natural stimuli (mouse urine) and monomolecular bile acid ligands, and with both somatic Ca^2+^ and spike frequency measurements. These results are not incompatible with previous observations that VSNs are spontaneously active, show bursting behavior in spontaneous and stimulated conditions, or that rate adaptation is mild during individual stimulus trials (Holy et al., 2000; Nodari et al., 2008; Arnson and Holy, 2011). These findings do, on the other hand, unambiguously show that short-term adaptation occurs when VSNs are stimulated repeatedly for 5 s or longer with ISIs <30 s.

Short-term VSN sensory adaptation was reversible over the time course of minutes. This was seen in VNO slices when Ca^2+^ signal adaptation to a brief accelerating ISIs was reversed after a ≥ 3 min recovery period (Fig. 1). In intact VNO epithelia, fast VSN adaptation steadily decreased when ISIs were gradually lengthened (decelerating ISI protocol; Fig. 4). Finally, VSN somatic spike rate adaptation was not observed when paired-pulse trials were separated by 2 min. We conclude that, whereas short-term VSN adaptation is severe at ISIs <30 s, these effects are reversible over the time course of minutes.

Population Ca^2+^ imaging experiments using monomolecular ligands revealed cross-desensitization in VSNs that were sensitive to both of the bile acid ligands (CA+DCA VSNs; Figs. 3, 4). In the absence of a recovery period >30 s, CA+DCA neurons that had adapted to DCA showed minimal initial responses to CA (Fig. 4). These data have important implications for the design of experiments aimed at assessing VSN tuning. For example, exposure to a panel of strong AOS ligands, even when using randomized, interleaved block designs (Nodari et al., 2008; Meeks et al., 2010; Turaga and Holy, 2012; Hammen et al., 2014; Xu et al., 2016), with ISIs <30 s may introduce stimulus “order effects,” increasing the likelihood of false negative (Type II) errors.

In addition to these technical considerations, these demonstrations of short-term VSN adaptation have cell physiologic implications. In line with previous observations (Spehr et al., 2009), adaptation of sensory responses provides a powerful mechanism for feedback regulation of vomeronasal sensitivity. One underlying mechanism is likely based on Ca^2+^/calmodulin-dependent feedback inhibition of primary VSN transduction channel(s) (Spehr et al., 2009). This model bears striking similarity to well-established sensory neuron adaptation in the MOS (Kurahashi and Menini, 1997; Munger et al., 2001; Song et al., 2008; Stephan et al., 2011). In canonical olfactory neurons, Ca^2+^/calmodulin-mediated shifts in CNG channel sensitivity effectively extend the dynamic range of signal detection. Similar gain control might be of particular physiologic importance in VSNs since these neurons are extremely sensitive to stimulation, with few picoamperes of depolarizing current being sufficient to elicit repetitive action potential discharge (Liman and Corey, 1996; Shimazaki et al., 2006; Hagendorf et al., 2009).

### Long-term VSN adaptation occurs over the course of tens of minutes and is stimulus timing dependent

Activation of VSNs is thought to be intrinsically slow due to the hydrophobicity of many AOS ligands and their need to be physically transported from the nasal cavity into the VNO lumen via vomeronasal mucus (Meredith and O’Connell, 1979). As such, natural sampling by the VNO may or may not normally induce the same short-term VSN adaptation revealed by these experiments. The long experimental durations of the population VSN Ca^2+^ imaging studies revealed an additional mode of cumulative VSN adaptation with a much slower time course. This slow, cumulative adaptation was prominent in the accelerating ISI protocol (Figs. 2, 3). When ISIs were gradually reduced over the course of 90 min, there was often little adaptation within a stimulus block but substantial adaptation across blocks. Eventually, the cumulative adaptation resulted in near-complete elimination of stimulus-driven GCaMP6s Ca^2+^ signals.

This slow adaptation seems to be linked to the specific ISI order, rather than being a simple consequence of repeated ligand exposure, photobleaching, or phototoxicity, because this type of adaptation was minimal in the decelerating ISI protocol. It is possible that accelerating ISI experiments preferentially caused phototoxicity, but this seems unlikely given that all experiments used the same imaging conditions (illumination power, acquisition rate, total imaging duration). Moreover, if phototoxicity were a function of stimulation (GCaMP6s brightness), one might expect the time course of slow adaptation to be greater in CA+DCA VSNs, which were stimulated twice as frequently as other VSN types, but this was not found to be the case (Fig. 3). It is important to note that slow adaptation was only observed via GCaMP6s Ca^2+^ imaging in response to monomolecular bile acid ligands; it may be that adaptation is less prominent (or absent) in other experimental conditions.

Despite these caveats, the time course of slow adaptation more closely matches mouse behavioral paradigms that involve regular physical interaction with other animals and/or their social odors over the same time scale. In social settings, mice sporadically but continuously sample environmental ligands and each other, which is likely to “pulse” the VNO with AOS chemosignals (Luo et al., 2003). If the same complement of environmental ligands is continuously detected (for example, when a mouse is introduced into the cage or habitat of another mouse), slow adaptation may gradually reduce VSN sensitivity to AOS ligands.

### VSN adaptation is non-uniform and is more pronounced for same sex than for opposite-sex cues

Most VSNs demonstrated short-term adaptation to urinary cues, but a substantial minority of these neurons failed to adapt (Figs. 1, 6, 7). This reveals the presence of short-term-adaptation-resistant subsets of urine-responsive VSNs. Constituents of male and female mouse urine activate partially-overlapping sets of AOS neurons (Holy et al., 2000; He et al., 2008; Hendrickson et al., 2008; Ben-Shaul et al., 2010; Tolokh et al., 2013); the heterogeneity in VSN adaptation to urine may partially reflect the activation of VSNs expressing different receptors with different sensitivities to urinary cues. Moreover, recent studies suggest that long-term environmental exposure to AOS steroid ligands can selectively modify VSN sensitivity (Xu et al., 2016), and others identified sex-specific modulation of VSN sensitivity to urinary proteins (Dey et al., 2015). All these data show that VSN tuning can be modulated by chemosensory experience and host-specific factors (e.g., internal hormonal state), but it was not clear whether such phenomena extended to the types of shorter-term adaptation explored here.

In contrast to the confocal Ca^2+^ imaging and electrophysiological experiments, volumetric GCaMP6s imaging experiments did not identify substantial VSN populations that were resistant to short-term adaptation. An important difference between these groups of experiments is that confocal Ca^2+^ imaging and electrophysiological experiments each used dilute mouse urine as sensory stimuli, whereas volumetric GCaMP6s Ca^2+^ imaging experiments used monomolecular bile acid ligands at 10 µM. The lack of a non-adapting population in volumetric imaging experiments may, at least in part, reflect the strong concentration of the bile acid ligands used. However, it is also possible that prior environmental sensory experience (i.e., to cues continuously present in the housing environment) may also be a factor. The two bile acids used, CA and DCA, happen to be common fecal components that are found at high levels in both male and female mouse feces (Doyle et al., 2016).

Further supporting the hypothesis that adaptation is modulated by prior chemosensory experience are the confocal Ca^2+^ imaging results, which revealed that VSN adaptation at intermediate ISIs was seen for same-sex urine but not opposite-sex urine (Fig. 7). Since mice were housed in same-sex cages, male VNOs would have only been exposed to male urine in the weeks before experiments; vice versa for females. Therefore, these data suggest that VSNs’ capacity for adaptation may depend on recent exposure to urinary cues. Urinary AOS ligands include, but are not limited to, sulfated steroids (Nodari et al., 2008), major urinary proteins (MUPs; Chamero et al., 2007) and major histocompatibility complex (MHC) peptide ligands (Leinders-Zufall et al., 2004), so it remains unclear whether the capacity to adaptation depends on molecular classes or cognate VSN chemoreceptors.

Overall, these data provide extensive evidence for VSN adaptation to natural ligand blends and monomolecular ligands on physiologically relevant time scales. Together, these studies provide a framework for future work investigating the molecular and cellular underpinnings of vomeronasal sensory adaptation and its impacts on AOS-mediated behavior.

## Synthesis

Reviewing Editor: Zoltan Nusser, Institute of Experimental Medicine Decisions are customarily a result of the Reviewing Editor and the peer reviewers coming together and discussing their recommendations until a consensus is reached. When revisions are invited, a fact-based synthesis statement explaining their decision and outlining what is needed to prepare a revision will be listed below. The following reviewer(s) agreed to reveal their identity: Haiqing Zhao.

The reviewers and I found that the experiments were well designed and rigorously conducted. The chosen stimulation paradigms were well justified. The results were clear and convincing. The finding that the majority of VSNs are similar to most of other sensory receptor cells to exhibit short-term adaptation is highly significant. The finding about the slow, cumulative adaptation phenomenon and the sex-dependent response difference are also interesting.

There are only few minor issues, the authors need to address prior to publications.

There is little to criticize in this manuscript. The experimental rationale and design are clearly described and properly controlled, and results conservatively interpreted. Importantly, the authors query the system with both complex and monomolecular stimuli, and use both Ca2+ imaging and electrophysiological approaches to validate their findings. The observation that adaptation to some stimuli was sexually dimorphic is an intriguing one that will surely be followed up on by the field.

A minor issue is the diminutive size of Figure 5. Both the rasters and some of the fonts are too small to see clearly. The entire figure should be enlarged.

Panel labels if Figures 5 and 6 should be bolded to match the other figures.

The authors showed in the conventional dye-based calcium imaging experiments (Figure 1) that a small population of VSNs did not show adaptation. I am wondering whether this lack of adaptation would be a true property of these VSNs, i.e. these VSNs do not adapt, or this would merely represent some specific situation of the experiments, e.g. less ideal ligands, too long of inter-stimulation intervals etc. The neuron in Figure 1 that did not show adaptation had smallest initial response amplitude. Did the volumetric imaging (though different ligands) and electrophysiological recordings (though low number of cells) find similar results?

1. Line 7, Abstract. Delete “by three collaborating laboratories”

2. Line 82, Methods. “Final DMSO concentrations were {less than or equal to} 0.1%.” Where is the DMSO used in the experiments?

3. Line 90. What is the final methanol concentration in CA, DCA stimuli? Did the perfusion solution contain the same amount of methanol?

4. Figure 2, 3 and related text. What is the inter-block interval for these experiments? How would the inter-block interval influence the initial response of the subsequent block?
